# School Nurse Perceptions of Their Role, Burnout, and Mentorship Programs: A Qualitative Analysis

**DOI:** 10.1177/10598405251371766

**Published:** 2025-08-26

**Authors:** Meghan L. Shah-Hartman, Katie E. Greenawalt, Erika VanDyke, Alicia M. Hoke, Deepa L. Sekhar

**Affiliations:** 1 Department of Pediatrics, 12310 Penn State College of Medicine, Hershey, PA, USA; 2 Department of Medicine, 12310 Penn State College of Medicine, Hershey, PA, USA

**Keywords:** mentoring, mental health, burnout, qualitative research

## Abstract

The COVID-19 pandemic exacerbated demanding workloads and poor working conditions for school nurses, both of which are strong predictors of burnout. This study explores Pennsylvania school nurses’ perspectives on burnout and the value of peer mentorship programs in mitigating burnout. Fourteen (N = 14) school nurses who served as mentors or mentees in a one-year (2023–2024) mentorship program participated in one of two focus groups. Content analysis was performed by two coders using MAXQDA software (Cohen's kappa coefficient = 0.75). Results demonstrate that school nurses feel misunderstood in their role; they additionally experience frequent worry, low availability of substitutes, high student caseloads, stress over managing multiple buildings, and challenges navigating administrative and parental relationships; these factors were identified as sources of burnout. Nurses view peer mentorship positively, valuing the opportunity to share ideas and experiences and benefiting from a supportive organizational culture. Qualitative data supports that peer mentorship may reduce school nurse burnout.

## Introduction

School nurses play an integral role in supporting student and school community wellness (National Association of School Nurses, 2024; Smith & Firmin, 2009; Willgerodt et al., 2024). School nurses have been referred to as experts in the domains of public health, pediatric, emergency, and mental health due to their many, varied roles, including education of students, advocating for student health, managing acute and chronic student medical conditions, communicating with parents and coordinating care, performing assessments, and preparing for emergencies (National Association of School Nurses, 2024; Smith & Firmin, 2009; Willgerodt et al., 2024).

A pre-pandemic study of United States (U.S.) school nurse burnout included cross-sectional data from 100 New Jersey school nurses (Jameson & Bowen, 2020). Burnout averaged in the moderate range on the validated Maslach Burnout Inventory. Higher scores for emotional exhaustion, the core component of burnout, were significantly associated with higher workloads and poorer work environments (Jameson & Bowen, 2020; Maslach et al., 1997).

Though the published literature on U.S. school nurse burnout during and post pandemic is scarce, the existing research has demonstrated that the COVID-19 pandemic precipitated U.S. nurse burnout and a national nursing workforce shortage (Murthy, 2022). School nurses were similarly impacted with an estimated 18% leaving their profession from 2018–2023 (Willgerodt et al., 2018; Willgerodt et al., 2024). U.S. school nurses were tasked with added responsibilities of COVID testing, results notification, and contact tracing (Merkle et al., 2022). Data indicates that 48% of school nurses reported bullying, threats, harassment, stigma, and discrimination during the pandemic (Merkle et al., 2022). Despite the shifting, growing responsibilities of school nurse practice, school nurse compensation has remained lower than their licensed counterparts working in hospital and outpatient settings with significant variability in school nurse staffing and caseload by geography (Hoskote et al., 2023; Jean, 2022; Lawson et al., 2021; Willgerodt et al., 2024).

Peer mentorship is an organizational strategy successfully used to reduce burnout among hospital-based nurses, demonstrated through systematic reviews, individual studies, and less formal program evaluations both inside and outside of the US with outcomes evaluations at eight weeks and up to three years (Brook et al., 2019; Cavanaugh et al., 2022; Chen & Lou, 2014; Gill-Bonanca, 2024; Glassman, 2020; Gularte-Rinaldo et al., 2023; Lang et al., 2023; Mínguez Moreno et al., 2023; National Academies of Sciences, Engineering, and Medicine, 2019; Ohue & Menta, 2024; Pham et al., 2019; Szalmasagi, 2018; Temkar, 2020; Zhang et al., 2019). However, data specific to school nurse mentorship and burnout remains limited. Literature reviews reveal that several states, including Pennsylvania, have successfully run school nurse peer mentorship programs (Gavin & Pleus, 2024; Hoke et al., 2024; Nebraska Department of Education, 2025; Sperry & Yonkaitis, 2024). However, corresponding data on the use of peer mentorship to reduce school nurse burnout and rigorous assessment and evaluation of these programs are lacking.

The goals of this study were to use focus groups to explore school nurses’ perceptions of their roles in relation to their initial expectations, role-specific challenges expressed in their own terms, and opinions on the potential of a year-long Pennsylvania school nurse peer mentorship program to reduce burnout (Hoke et al., 2024). Through our program and focus group interviews, we aimed to gather additional data on peer mentorship and school nurse burnout to build on current research gaps. Additional goals of our pilot mentorship program can be found in Hoke et al. (2024).

## Methods

### PA Pilot Mentorship Programming and Participant Background

In the state of Pennsylvania, all school nurses are required to have a school nurse certification. A Pennsylvania certified school nurse (CSN) is defined as an individual with Registered Nurse (RN) licensure who is certified by the Superintendent of Public Institution as a school nurse (Pennsylvania General Assembly, 1949). In Pennsylvania, public schools may request an Emergency Permit for individuals who have not yet obtained a School Nurse Certification but are actively working toward the required education. These permits allow candidates to serve as school nurses while completing the necessary coursework and qualifications (Commonwealth of Pennsylvania, 2024). During the 2023/2024 school year, Penn State PRO Wellness launched a school nurse mentorship program with 30 Pennsylvania school nurses (15 mentors, 15 mentees). PRO (Prevention, Research, Outreach) Wellness is a group within the Department of Pediatrics within Pennsylvania State College of Medicine that runs a wide variety of community-focused research and educational programming and has a history of partnership with schools and school nurses dating back to 2003.

Mentees were required to hold either a bachelor's degree in nursing or a bachelor's degree in another field with unrestricted RN licensure, and meet one of the following inclusion criteria: (1) currently pursuing their PA emergency CSN certification, (2) graduated from a CSN program within the last five years, or (3) were employed as a CSN with less than five years of experience. Eligible mentors were either a current CSN with at least five years of experience within the school setting or a CSN who had retired within the last three years (Hoke et al., 2024).

The school nurse mentorship program included two components: (1) one-to-one mentorship meetings guided by a co-developed meeting plan and (2) a five-part professional learning seminar series (four virtual, one in person) designed to complement mentorship-supported learning by covering timely topics in school nursing (Hoke et al., 2024). Seminar topics included mild traumatic brain injury, prioritizing mental health, autism, health promotion and prevention beyond the health suite, and tips for mandated health screenings (Hoke et al., 2024). Mentored pairs were expected to meet five times between November 2023 and April 2024, using the modality of their choice (e.g., virtual or in person). Pairs were asked to develop a plan to guide the topics of one-to-one mentorship meetings. Topics were based on the five topics from the 21st Century School Nursing Practice™ Framework (National Association of School Nurses et al., 2015). The framework has since been updated (National Association of School Nurses et al., 2024). Each mentored meeting focused on one of five topics that mentees identified as areas for mentorship or improvement (Hoke et al., 2024).

Program evaluations completed by participants report high fidelity to mentor/mentee meeting attendance requirements and high levels of satisfaction with their experience. Additionally, after participating in the program, mentees reported increased confidence in addressing topics discussed during mentored meetings. Further details of the evaluation results from the pilot mentorship program can be found in Hoke et al. (2024). This current study seeks to further explore participants’ perceptions of the program and gain a deeper understanding of attitudes toward the school nursing profession in relation to initial expectations, with particular attention to challenges specific to the school nurse role and how peer mentorship may positively influence experiences of burnout.

### Qualitative Study Design

Using an exploratory qualitative design, two focus groups were conducted with a semi-structured focus group guide (Krueger & Casey, 2002). The focus groups were conducted at the statewide annual meeting of the Pennsylvania Association of School Nurses and Practitioners (PASNAP) (March 23, 2024, Hershey, Pennsylvania). Thirty-minute focus groups were facilitated by two members of the mentorship program team; these members were selected due to prior experience conducting focus group interviews. Following introductions and consent, the focus groups were audio recorded to facilitate transcription. Participants were assigned a number and advised to refer to each other by number versus name to support anonymity of the transcription. The study was reviewed and approved by the Pennsylvania State College of Medicine's Institutional Review Board (STUDY00024637).

### Participants

Mentorship program participants (N = 30) were encouraged to attend the PASNAP annual conference to meet other participants and participate in the final in-person seminar with cost of the meeting registration covered by the program. Participants attending the PASNAP conference were invited via email to participate in a focus group. As such, this sample represents a convenience sample of participants who were able to attend the PASNAP conference (N = 14). Participants were compensated with a $20 e-gift card for their participation.

### Procedures and Instruments

Participants were divided into two focus groups to foster an environment that encouraged active participation and ensured that each nurse had the opportunity to contribute. Each group was mixed, containing both mentors and mentees. Two study team members facilitated each focus group. One team member used a semi-structured focus group guide to lead the discussion (Table 1). The other managed the audio recording. The focus group guide elicited discussion of various topics, including participants’ decisions to pursue school nursing, their professional challenges, their experiences with the mentorship program, and burnout. Discussions were audio-recorded and professionally transcribed by Rev.com.

**Table 1.** Focus Group Interview Questions.

Question number	Question
1	How did you decide to pursue school nursing and how many years have you been in the profession?
2	Is being a school nurse what you envisioned (e.g., hours, caseload, student medical complexity)?
3	What are the most challenging aspects of being a school nurse?
4	Burnout is defined as a state of mental, emotional and physical exhaustion resulting from repeated stress. There is a large amount of literature documenting nurse burnout, primarily clinical nurses in the hospital setting, and especially post-pandemic. Can you share your insights on burnout from a school nurse's perspective?
5	What made you sign up to be a mentor or mentee in this program?
6	Do you feel the school nurse mentorship program impacted school nurse burnout? If so, how?
7	What adjustments to the program might help nurses combat burnout?
8	Is there anything else you would like to share?

### Qualitative Analysis

Data from focus group transcripts were analyzed using descriptive content analysis (Saldana, 2011). Two analysts reviewed the transcripts and inductively generated codes. A preliminary codebook was developed, and the codebook was reviewed and agreed upon by several members of the research team. A final codebook was developed, which included definitions for each code to improve consistency among coders. Using the codebook (Table 2) and the MAXQDA software (Version 24), the analysts independently coded data from each transcript. The coded transcripts achieved a Cohen's kappa coefficient of 0.75, reflecting a substantial level of inter-rater agreement, as per established guidelines (McHugh, 2012). While data saturation typically guides sample size in qualitative research, our study included the sample of PA Mentorship Program participants who were able to attend the in-person statewide annual PASNAP meeting (N = 14). Rather than sampling until no new themes emerged, we conducted comprehensive focus groups with all available participants to capture the full range of experiences within this bounded program. Our content analysis revealed consistent themes across groups, suggesting adequate thematic coverage despite the predetermined sample size. This approach aligns with research contexts where the population of interest is naturally limited by program participation (Palinkas et al., 2015). Consolidated Criteria for Reporting Qualitative Research (COREQ) guidelines for rigor were followed in the reporting of these results (Tong et al., 2007).

**Table 2.** Focus Groups Codebook (Inclusive of Categories, Codes, and Definitions).

Category	Code	Definition
1. Participant Nursing Background		Code to be used for anytime a nurse provides a history or background of his/her nursing career (question 1, code each background separately)
		
2. Overall Perception of School Nurse Role		
	2a. Nothing can prepare you	The feeling that nothing can prepare you for the role, no amount of schooling or certificate program can prepare you for taking on this role, role is intense
	2b. Need assessment skills	To be a successful nurse, you must have sharp assessment skills to deal with complex cases
	2c. Important provider role	Nurses describe their role is important because they are the only healthcare provider for the school, sometimes only provider that the student has seen in a while, their role is to provide care to children
	2d. Leader in school	In their role, nurses must be autonomous and lead in the school, need to garner respect from admin
	2e. Need to be flexible	Role requires flexibility because every day throws curveballs
	2f. Perception is not what others think	Many people think nursing is slow and boring, but their perception is their job is anything but boring; their job is not what others think.
		
3. School Nurse Burnout and Challenges		
	3a. COVID related factors	Stressful time from COVID, use code anytime they talk about COVID.
	3b. Constant worry	Unlike bedside nursing, they cannot leave work at work, they take their cases home with them, connections make it hard to “unplug,” constantly worrying about kids, critical of oneself and worrying about decisions.
	3c. No substitutes	No one can cover a nurse if they need a day off, they cannot miss a day, leading to stress, they are the only person with medical background in the school
	3d. One nurse for many buildings	When one nurse is managing multiple buildings and cannot be in multiple places at once, one nurse for too many students, census too high
	3e. Difficult student cases	Managing some student cases is difficult stemming from lack of follow-up with parents, difficult cases, treating in the school setting
	3f. Navigating parent interactions	Dealing with difficult parents leads to stress
	3g. No support	Feels they do not have supportive admin, no supportive staff, no structural support, psychological support, etc.
		
4. Benefits of Program		
	4a. Ability to share ideas	Liked being able to bounce ideas off someone, talk about things with someone who understands, get different perspectives, problem solve
	4b. Wants program to continue	Nurses want program to continue, recognize the importance of the program
	4c. Comradery	To be used when nurses highlight the friendship and relationship formation as a benefit of the program
	4d. Psychological Safety	To be used when nurses note feeling supported for their challenging role, that the environment was safe, no judgement, more of a focus on psychological safety
		
5. Resources Available in Their School		To be used anytime a nurse talks about the current resources they have available to them in their school

## Results

### Participant Characteristics

Nearly half (n = 14) of the mentorship program participants (mentees = 7; mentors = 7) attended the PASNAP annual conference and all were invited and agreed to participate in one of two focus group interviews. All participants identified as non-Hispanic, white females.

### Content Analysis

Five themes were identified related to school nurse roles being misunderstood, the emotional investment of school nursing, serving as the sole medical professional, challenging parent interactions, and mentorship program benefits.

**Theme 1: School nurses often find their role in student health and well-being undervalued and misunderstood by education colleagues.**

School nurses describe playing a crucial role in the health and well-being of students, yet expressed that “no one knows what we really go through.” Nurses expressed frustration at the widespread misconceptions about their responsibilities, particularly from administrators and parents who fail to understand the complexity of their role. Nurses described feeling particularly misunderstood during their transition from hospital-based roles. As one nurse explained:

*When I went into school nursing when I was leaving the hospital, I can't tell you how many people said to me like, ‘Oh, make sure you bring books to read. If you sew, bring your quilt. You're going to have extra time to do it.’ And it's so, so different than that. You're more than the nurse.—Focus Group 2, Speaker #7*

Beyond being dismissed, some nurses found others failed to grasp the level of clinical acumen that the broad scope of their roles demanded. This nurse emphasized the depth of clinical knowledge required to work in a school setting:

*I think also some people's perceptions of what a school nurse is and our knowledge base, I've had people say, ‘Are you a real nurse?’ Because they don't understand that I think working in a school system, you actually have to be on the top of your game all the time. You never know what's going to walk through your door. It could be anything.—Focus Group 2, Speaker #5*

Nurses also discussed the challenge of being the only medical authority in an education-centered environment:

*It's very difficult being the medical entity in an educational realm because we get it as nurses, but nobody else does.—Focus Group 1, Speaker #1*

**Theme 2: The emotional investment school nurses make in their students can lead to emotional stress and burnout.**

Participants described the emotional burden of school nursing as extending well beyond the school day. School nurses reported forming strong attachments to their students, which contributed to ongoing worry and stress outside of work hours that they can’t “turn off.” The difficulty of emotionally disconnecting from the role was a common theme, as nurses emphasized the depth of their concern for “[their] kids.”

*I definitely think there's a big part with the emotional aspect of burnout in the schools. We are very familiar with our students who we see every day. So, when you’re going home, you can’t just turn that worry off. If something happened during the day and I go home and I’m still worrying about that kid, did they get the care that they needed, is everything okay? And it starts to drain on you after a while, you can’t turn it off.—Focus Group 2, Speaker #8*

Nurses also shared the emotional stress of caring for students whose health outcomes were often shaped by forces beyond their control, as evidenced by this nurse:

*You send a kid home, something's wrong, you’re not getting that follow-up unless the parent wants to give it to you. So then you’re calling, ‘Well, hey, what’d the doctor say?’ ‘Oh, I had some results, but I don’t know what they are.’ But as a nurse, you’re dying to know because…* *‘Well, what was their levels?’ I had a girl that was passing out constantly. She's running track and I’m like, ‘Well, what were your labs?’ She's like, ‘I don’t know.’ And it kills you because you want to know.—Focus Group 1, Speaker #2*

**Theme 3: Being the sole medical professional in a school setting contributes to stress.**

Participants described the overwhelming responsibility of serving as the sole healthcare provider in their schools was a major source of stress. Nurses reported caring for hundreds, or even thousands, of students without additional medical support. The “lack of school nurses” (i.e., substitutes), particularly for nurses assigned to multiple buildings was reported as a contributor to both physical and emotional exhaustion, as reported by this nurse:

*I have…worked for 34 years. When I started, school nursing was doable. I had five schools. The ratio was 1,500 students, 34 years later, 1,500 students is way too many to carry. We have chronic diseases…there's no lunch, there's no break. It's constant. When you walk through that door, it starts and it doesn’t end when the kids go home because then the phone starts.—Focus Group 2, Speaker #4*

Another nurse described the stress associated with being responsible for multiple school buildings simultaneously.

*I still have trouble comprehending that you can go from building to building, that there's buildings without nurses. That will be a reason, if I ever…* *I don’t want to, but that would be a reason that I would actually [leave]…* *Because it's not safe. It's not safe. And you get told, ‘Oh, I’m being called for emergencies.’ You can’t be in two places at once.—Focus Group 1, Speaker #2*

Another participant shared how the onset of COVID-19 deepened the emotional and logistical burdens of the role:

*I was by myself during COVID, so I had grade six through 12, 1,350 kids who were [participating in] double sports in middle school and high school. So if there was a case of COVID and they had practice, the team was down, I was on the phone all day, probably up until 11:00 at night.—Focus Group 1, Speaker #1*

**Theme 4: Parent relationships contribute to school nurse stress.**

Participants shared that they frequently found themselves at the intersection of medical care and “parental coordination.” Participants expressed frustration when dealing with “difficult” parents who exhibited inconsistent engagement with their children's health needs, as cited by this nurse:

*Some parents are there for their child, they will come, they will pick them up, they will do what needs to be done, will take them to the doctor. There's some who are mad if you call, are mad if you don’t call, so you’re not sure what the right answer is… I find that the most difficult thing.—Focus Group 2, Speaker #6*

Participants also shared difficulties in communicating medical urgency to parents, as one nurse explained:

*Or the parents just kind of blow it off, ‘Yeah, she’ll be okay.’ No, no. You have to make sure she's okay. And then I’ll call them again and call them again and they then complain to the principal.—Focus Group 1, Speaker #3*

**Theme 5: The mentorship program addresses the stress and isolation inherent in school nursing by fostering collaborative relationships and enabling new nurses to exchange diverse perspectives and innovative solutions with their mentors.**

The mentorship program was widely regarded as an essential support system for school nurses, particularly those who were new to the role. Participants highlighted how the opportunity to connect with experienced mentors helped alleviate feelings of “being on an island” and uncertainty. The ability to seek guidance and “bounce ideas” off others was described as buffering against stress and isolation, two factors that may contribute to burnout, as described by this nurse:

*You could be the best nurse in the world. But until you’re in the thick of it and you can just bounce those ideas off of somebody who's been in it, there's no book on that. You can’t Google something like that. Talking to somebody who's been in it, a mentor, this was a fantastic program and I think it would be so beneficial.—Focus Group 1, Speaker #2*

Another participant discussed how the program provided essential guidance and support:

*My first day in my office, I was alone. My first day as a school nurse, I was alone. I was put in that office, ‘This is your office.’ I went in a week before school started, saw where everything was, told, ‘This is the computer system.’ I had phone a friend, I did have a mentor at school… So this is really a great opportunity to get a different perspective.—Focus Group 1, Speaker #7*

## Discussion

This study highlights the persistent challenges faced by Pennsylvania school nurses, particularly the undervaluation of their roles and the potential for burnout due to the significant emotional and professional demands of their work. However, results also raise consideration of the promise of peer mentorship to mitigate stress and ultimately, burnout. These findings set the stage to progress to quantitative measures of burnout using validated tools. In addition, there is potential to apply the peer nurse mentorship model across states, and the opportunity to gather data on longer-term impacts of mentorship both for school nurses and the students they support.

Data from school nurse peer mentorship programs in Nebraska, Illinois, and New Jersey during/post-pandemic echo those from our Pennsylvania program in emphasizing the value of peer connection (Gavin & Pleus, 2024; Hoke et al., 2024; Sperry & Yonkaitis, 2024). School nurses were struggling in the wake of the COVID-19 pandemic, and while each state's school nurse mentorship program ran slightly differently in terms of meeting frequency and ratios of mentors to mentees, the goals were to better manage stress, reduce isolation, and create a supportive working environment (Gavin & Pleus, 2024; Hoke et al., 2024; Nebraska Department of Education, 2025; Sperry & Yonkaitis, 2024). The term burnout is not specifically used, but testimonials from the New Jersey program confirm the value of peer support and a positive culture (Gavin & Pleus, 2024). Similarly, Illinois’ program implemented during the 2022–2023 school year included 34 nurses who demonstrated increased knowledge on nursing topics but also highlighted the value of peer connection (Sperry & Yonkaitis, 2024).

As a systems level intervention, peer mentorship may be better positioned to address burnout compared to professional development or continuing education, as burnout is a direct result of organizational, versus individual-level, factors (Andersen & Watkins, 2018; Krishna et al., 2024; National Academies of Sciences, Engineering, and Medicine, 2019). These qualitative data position us to consider more robust evaluation using burnout-specific metrics. For example, the Maslach Burnout Inventory is a well-established assessment that has previously been used successfully with school nurses (Jameson & Bowen, 2020; Maslach et al., 1997). It could easily be integrated into the mentee and mentor baseline and one-year evaluation metrics of our Pennsylvania mentorship program, though mentorship impact on burnout may need to be assessed over a longer time period.

In addition, the opportunity exists to gather these data from multiple states to understand how results may be influenced by variations in school nurse credentialing, staffing, and scope of practice. There is also limited understanding of how long a mentorship relationship should continue to see success both as it relates to burnout and other benefits (e.g., improved peer connection, reduced stress, reduced isolation). At the conclusion of our one-year Pennsylvania program, 93% of mentees (14/15) indicated they would continue to stay in touch with their mentor (Hoke et al., 2024). A California nurse mentorship program that was not specific to school nurses found the benefits of mentoring are the greatest between one and two years and steadily increase up to two years of mentorship in the mentee (Gularte-Rinaldo et al., 2023). Consideration should be given to extending the collection of metrics over a longer time period.

Finally, the ultimate issue with school nurse burnout and turnover is that school nurses are integral to the health of our students, schools, and communities (Smith & Firmin, 2009; Willgerodt et al., 2024). Over 40% of today's students attend school with a chronic medical condition, and the absence of a school nurse impacts student chronic absenteeism (Centers for Disease Control, 2021; Jacobsen et al., 2016). For example, the reintroduction of school nurses into nine rural Michigan schools decreased student chronic absenteeism from approximately 27% to 18% over 5 years (Jacobsen et al., 2016). Thus, consideration should also be given to measuring student-related metrics as part of the long-term benefits of school nurse peer mentorship.

## Limitations

The study has several limitations that should be considered. First, the small sample size should be acknowledged; only half the mentors and mentees in the program were able to participate in the focus groups, and specific demographic data (aside from 100% of participants identifying as non-Hispanic white females) for the 14 participating school nurses were not recorded. Their perspectives may differ from those who were not able to attend. Another limitation to consider is that while the focus groups were deidentified to promote honesty and confidentiality, there is no way to determine who was a mentee versus a mentor after the transcripts were coded; there may be differences in responses based on what role the participant held. Additionally, while the study focused on the experiences of Pennsylvania school nurses, their perspectives do not fully represent the experiences of school nurses nationally due to regional differences in resources, policies, and challenges. Selection bias must also be acknowledged, as nurses selected themselves as participants in the study. Another consideration is courtesy bias; although the school nurses were encouraged to be honest with their feedback, the fact that the focus groups were facilitated by program staff and included fellow school nurses may have made participants hesitant to disclose negative feelings about the program or their mentor/mentee experiences (Hameed et al., 2018). To overcome these limitations, subsequent studies should include multiple focus groups with larger, more diverse samples to capture a broader range of perspectives and experiences. Future studies should also consider the use of external evaluators to avoid courtesy bias.

## Implications for School Nursing Practice and Conclusions

The findings from this qualitative study of participants in the Pennsylvania school nurse peer mentorship program suggest that school nurses may be at risk for burnout due to multifactorial circumstances including demanding workloads, lack of understanding of their roles, and stress/worry regarding student health. Peer mentorship is a promising approach to build a supportive organizational culture and potentially address burnout. These data set the stage to consider quantitative measures of burnout in the context of school nurse peer mentorship programs as well as long-term data collection for both school nurse and student outcomes. Future studies should build on these findings to create a conceptual framework of burnout that is specific to school nursing, as similar frameworks exist for educators and clinicians that would be beneficial to expand into the nursing realm (Chang, 2013). Ultimately, school nurse burnout is important to address not only for the health of our nurses, but also for downstream effects on student health (e.g., chronic absenteeism) (Jacobsen et al., 2016; Rodriguez et al., 2013; Telljohann et al., 2004).
